# Beta-cell hubs maintain Ca^2+^ oscillations in human and mouse islet simulations

**DOI:** 10.1080/19382014.2018.1493316

**Published:** 2018-08-24

**Authors:** Chon-Lok Lei, Joely A. Kellard, Manami Hara, James D. Johnson, Blanca Rodriguez, Linford J.B. Briant

**Affiliations:** a Doctoral Training Centre, University of Oxford, Oxford, UK; b Department of Computer Science, University of Oxford, Oxford, UK; c Oxford Centre for Diabetes, Endocrinology, and Metabolism, Radcliffe Department of Medicine, University of Oxford, Churchill Hospital, Oxford, UK; d Department of Medicine, The University of Chicago, Chicago, USA; e Department of Cellular and Physiological Sciences, Diabetes Research Group, Life Sciences Institute, University of British Columbia, Vancouver, Canada

**Keywords:** GJ, gap junction, [*Ca*^2+^]*_i_*, intracellular calcium concentration, T2DM, type 2 diabetes mellitus, *V_m_*, membrane potential, GCK, glucokinase, SERCA, sarcoplasmic reticulum Ca^2+^-ATPase

## Abstract

Islet β-cells are responsible for secreting all circulating insulin in response to rising plasma glucose concentrations. These cells are a phenotypically diverse population that express great functional heterogeneity. In mice, certain β-cells (termed ‘hubs’) have been shown to be crucial for dictating the islet response to high glucose, with inhibition of these hub cells abolishing the coordinated Ca^2+^ oscillations necessary for driving insulin secretion. These β-cell hubs were found to be highly metabolic and susceptible to pro-inflammatory and glucolipotoxic insults. In this study, we explored the importance of hub cells in human by constructing mathematical models of Ca^2+^ activity in human islets. Our simulations revealed that hubs dictate the coordinated Ca^2+^ response in both mouse and human islets; silencing a small proportion of hubs abolished whole-islet Ca^2+^ activity. We also observed that if hubs are assumed to be preferentially gap junction coupled, then the simulations better adhere to the available experimental data. Our simulations of 16 size-matched mouse and human islet architectures revealed that there are species differences in the role of hubs; Ca^2+^ activity in human islets was more vulnerable to hub inhibition than mouse islets. These simulation results not only substantiate the existence of β-cell hubs, but also suggest that hubs may be favorably coupled in the electrical and metabolic network of the islet, and that targeted destruction of these cells would greatly impair human islet function.

## Introduction

Pancreatic β-cells have a central role in type 2 diabetes mellitus (T2DM) pathophysiology.^1,2^ These islet cells are responsible for secreting insulin in response to rising plasma glucose concentrations. Robust, islet-wide oscillations in intracellular Ca^2+^ are required for glucose-stimulated insulin secretion.^3,4^ These oscillations are highly synchronized, due to gap junction (GJ) coupling between β-cells.^5-7^ The nature of these oscillations depends on the proliferative,^8^ developmental^9,10^ and differentiated^11,12^ state of the cell. Multiple animal models of diabetes have also demonstrated that the impaired insulin secretion characteristic of this disease, is due, in part, to dysfunctional Ca^2+^ oscillations,^13-18^ with studies on human β-cells corroborating this finding.^19-21^ These Ca^2+^ oscillations drive pulsatile insulin release^22^ – a secretory pattern that enhances hepatic insulin action,^23^ protects against insulin resistance,^24^ and is lost in T2DM.^25-28^ To understand how these Ca^2+^ oscillations become defective in T2DM, it is important to first understand how an islet generates and maintains these oscillations.

In the heart, the sinoatrial node contains specialized myocytes that coordinate the Ca^2+^ waves necessary for initiating a cardiac cycle.^29^ A similar system has long been postulated to exist in the islet, whereby specialized β-cells generate and pace the Ca^2+^ oscillations necessary for insulin secretion.^30-32^ Recently, Johnston *et* *al*.^33^ demonstrated, by using functional cell mapping and optogenetics, that certain β-cells (termed ‘hubs’) are indispensable for the maintenance of Ca^2+^ activity in the islet. Silencing of these cells revealed that inhibition of a single hub cell could reduce Ca^2+^ activity in the islet network. These cells constitute 1-10% of the islet; therefore, the activity of the islet is highly dependent on a small proportion of β-cells. They reported that these cells: are highly metabolic, due to high glucokinase protein (GCK) expression; have reduced expression of sarcoplasmic reticulum Ca^2+^/ATPase (SERCA2) and insulin content; and are transcriptionally ‘immature’ due to the low expression levels of signature β-cell transcription factors (e.g. *Pdx1*). Such cells could therefore be more susceptible to both pro-inflammatory and glucolipotoxic insults in T2DM,^8,9,34,35^ which would ultimately result in whole-islet failure and impaired insulin secretion.

The findings of Johnston et al.^33^ are impressive and convincing, but, like all studies, their work was not without limitations. Their imaging methodology consisted of recording Ca^2+^ oscillations in all β-cells in a 20 µm confocal plane. In a spherical islet, this would typically be the first two layers of cells on the surface of the islet, amounting to ~50-100 cells, or ~5-15% of all β-cells in the entire islet^36^; hence, the conclusions of the study are limited to the ‘imaged plane’, and do not extend to the whole islet. In particular, it is not clear if hub inhibition influences Ca^2+^ activity in the entire islet, or just Ca^2+^ activity in the imaged network. Secondly, to allow selective inhibition of identified hubs, Johnston et al.^33^ used a transgenic mouse line that expressed halorhodopsin in β-cells - an approach that would be difficult to implement in human islets. How, then, do these findings in mice translate to human islets? It is important to carefully consider this question, because mouse and human islets display different β-cell Ca^2+^ dynamics; mouse β-cells display islet-wide synchrony in response to glucose, whereas synchrony in human β-cells is constrained to localized subpopulations.^37^ These differences likely stem from the differences in human and mouse islet architectures: mouse islets have a highly connected β-cell core, whereas β-cells in human islets occur in distinct clusters.^38-40^

Computational modeling offers a suitable and valuable paradigm for investigating the role of hubs in human islets. In this study, we conducted parallelized simulations of computational models of mouse and human islets. These models displayed detailed morphological features based on 3D confocal reconstructions of islets.^36,41^ We then used these models to explore the experimental findings of Johnston et al.^33^ In particular, we ask the question: can endowing a model of a mouse islet with a few (10%) highly metabolic β-cells recapitulate the findings of Johnston et al.^33^? We explored this question in an impartial and objective manner by considering parameter uncertainty, cell-to-cell variability, repeating simulations for different random seeds and constructed models for a number of mouse and human islet architectures.

## Computational methods

### Model of *β*-cell membrane potential and intracellular Ca^2+^ dynamics

There are many models of β-cells, which have been reviewed by Pedersen.^42^ We used the ‘Cha-Noma model’^43^ because this model gives a detailed description of membrane potential ($V_{m})$ and intracellular Ca^2+^ $\left({\left[Ca^{2+}\right]}_{i}\right)$ dynamics. The underlying equations can be found therein. In brief, the model of β-cell $V_{m}$ is described by:

(1) $$\begin{array}{rl} & C_{m}\frac{dV_{m}}{dt}=-\left(I_{CaV}+I_{KRPM}+I_{SOC}+I_{bNSC}\right.\\ & \;\;\;\;\;+\;I_{KDr}+I_{KCa}+I_{KATP}+I_{NaK}\\ & \;\;\;\;\;\left.+\;I_{NaCa}+I_{PMCA}+I_{coup}+I_{NpHR}\right) \end{array}$$

where $C_{m}$ is the cell capacitance and $I_{X}$ is the electrical current due to channel type X. Full details of the functional forms and parameters for each of these currents can be found in Cha et al.^43^ Here, we have added two currents. $I_{NpHR}$ is the halorhodopsin (NpHR) current; this was employed by Johnston et al.^33^ to inhibit hub cells. $I_{coup}$ is the current due to GJ coupling of the β-cell with a spatially-contacting β-cell.

The equation describing ${\left[Ca^{2+}\right]}_{i}$ dynamics was:

(2) $$\frac{d{\left[Ca^{2+}\right]}_{i}}{dt}=-\frac{f}{v}\left(\frac{\sum I_{Ca}}{2F}-J_{SERCA}+J_{rel}\right)$$

where F is the Faraday constant, f is the cytosolic Ca^2+^ buffer strength and v is the cell volume. $\sum I_{Ca}$ is the total transmembrane Ca^2+^ current. Endoplasmic reticulum (ER) Ca^2+^ dynamics are also included, via the flux terms for uptake by the ER Ca^2+^-ATPase $\left(J_{SERCA}\right)$ and ER Ca^2+^ release $\left(J_{rel}\right)$.

The parameter values can be found in the model code. These were identical to the original model by Cha et al.^43^, aside from the modifications described in detail below.

### Spatial configuration of islet models

The 3D structures of 8 human (4 donors) and 8 mouse (4 mice) islets were provided from a previous study.^36^ These islets were size-matched on the number of β-cells across the species. For each islet structure, we constructed a mathematical model of the islet that included all the β-cells and the necessary GJ connections between these cells (S1 Figure). The methodology for this process has previously been described in detail.^41^ The experimental dataset provided the $\left(x,y,z\right)$ coordinates of the DAPI-stained nucleus of each insulin^+^ cell in the islet; namely, the spatial location of each β-cell in the islet. The Cha-Noma model of a β-cell was then placed at the $\left(x,y,z\right)$ location of each β-cell. What remains to be determined is which cells are in spatial contact with one another, and therefore form functional (e.g. GJ) connections.

Two β-cells, with coordinates $X_{1}=\left(x_{1},y_{1},z_{1}\right)$ and $X_{2}=\left(x_{2},y_{2},z_{2}\right)$, were considered to be spatially in contact if

(3) $$||X_{1}-X_{2}||<d_{thr}$$

where ||⋅|| is the Euclidean distance and $d_{thr}=17.5$ µm. This threshold distance was selected because (a) it is approximately the diameter of a β-cell (~10-12 µm^44,45^) and (b) it yields on average 8-10 spatial contacts per cell, which lies within the number of contacts according to the thinnest (6 contacts) and densest (12 contacts) regular sphere packing algorithm for spheres of diameter 12 µm. For each islet, we computed the number of spatial contacts for each β-cell in the islet, and generated a histogram of these data for that islet.

### Determining gap junction connections in islet model

If two β-cells were deemed spatially in contact, a non-zero GJ conductance was assigned to electrically couple them. The GJ conductance was picked from a Gaussian distribution with mean μ=50 pS and standard deviation of$\;\sigma =\left(0.7\mu \right)$ pS. This unitary strength is in good agreement with recordings in intact mouse islets (50–120 pS unitary strength^46^) Given that each β-cell in our mouse islet architectures had on average 10 GJ connections (Figure 5G), the total GJ conductance for each β-cell would range between 150 and 850 pS ($10\times \left(\mu \pm \sigma \right)$). This total GJ conductance is comparable to recordings from intact mouse islets (total GJ conductance 1200 pS.,^47^) These GJ conductance values are also in agreement with previous simulation studies in cubic clusters of mouse β-cells.^48^

**Figure 1.**
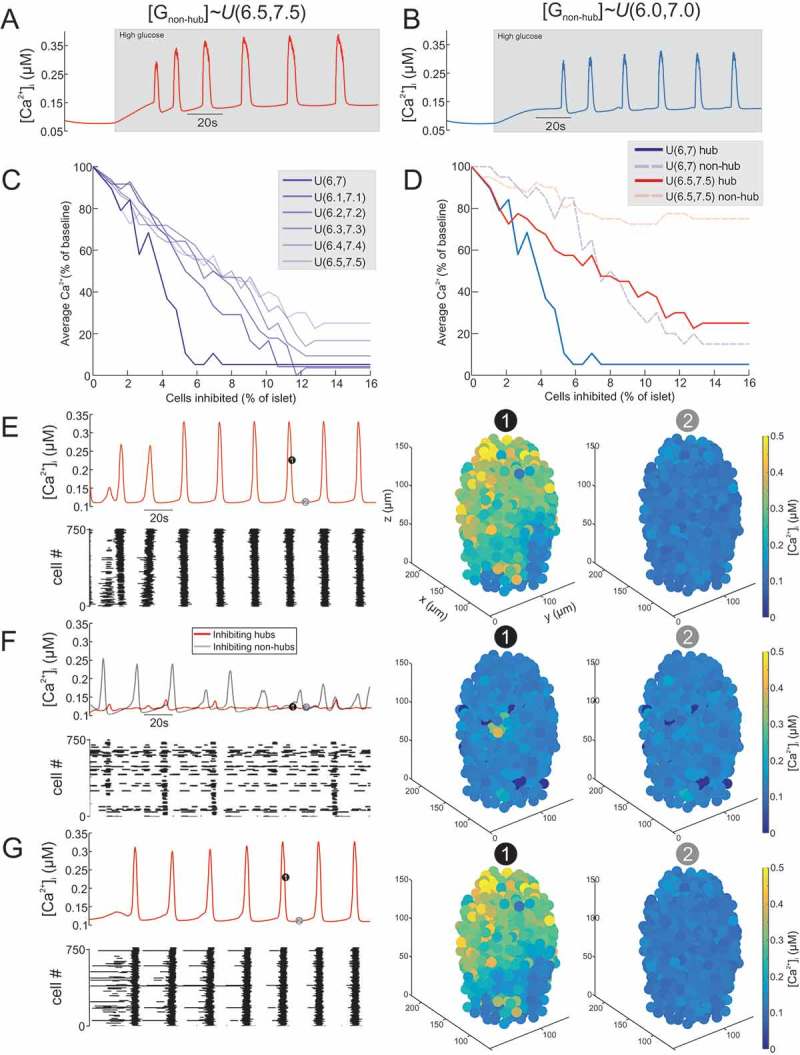
Inhibition of hub cells can abolish whole-islet Ca^2+^ activity. (A) ${\left[Ca^{2+}\right]}_{i}$ activity of mouse islet model when the GJ conductance for non-hubs $\left(\left[G_{non-hub}\right]\right)$ is sampled from a uniform distribution over the interval 6.5-7.5mM $(\left[G_{non-hub}\right]∼U\left(6.5,7.5\right)$). The model produces robust ${\left[Ca^{2+}\right]}_{i}$ oscillations in response to high glucose. (B) ${\left[Ca^{2+}\right]}_{i}$ activity of mouse islet model when the GJ conductance for non-hubs $\left(\left[G_{non-hub}\right]\right)$ is sampled from a uniform distribution over the interval 6.0-7.0mM $\left(\left[G_{non-hub}\right]∼U\left(6,7\right)\right).$ The model produces robust ${\left[Ca^{2+}\right]}_{i}$ oscillations in response to high glucose. Simulated islet (C) ${\left[Ca^{2+}\right]}_{i}$activity during inhibition of hub cells. The number of hub cells inhibited is represented as the % of all cells in the islet (750 cells). The different simulations are for sampling $\left[G_{non-hub}\right]$ from different uniform distributions. Note how hub inhibition has the strongest effect when $\left[G_{non-hub}\right]∼U\left(6,7\right)$. Simulated islet (D) ${\left[Ca^{2+}\right]}_{i}$ activity during inhibition of hub or non-hub cells. When $\left[G_{non-hub}\right]∼U\left(6,7\right)$ mM, hub inhibition strongly suppresses whole-islet ${\left[Ca^{2+}\right]}_{i}$. In contrast, when $\left[G_{non-hub}\right]∼U\left(6.5,7.5\right)$ mM, hub inhibition has little effect on whole-islet ${\left[Ca^{2+}\right]}_{i}$. Mean (E) ${\left[Ca^{2+}\right]}_{i}$ for all β-cells in a mouse islet model, during high glucose condition. Raster plot showing ${\left[Ca^{2+}\right]}_{i}$ activity in each β-cell. 3D plot of ${\left[Ca^{2+}\right]}_{i}$ for each β-cell in the islet model at time points (1) and (2). Mean (F) ${\left[Ca^{2+}\right]}_{i}$ for all β-cells in a mouse islet model, during hub inhibition and non-hub inhibition. 45 hub cells or non-hub cells where inhibited simultaneously. Raster plot showing ${\left[Ca^{2+}\right]}_{i}$ activity in each β-cell during the hub inhibition condition. 3D plot of ${\left[Ca^{2+}\right]}_{i}$ for each β-cell in the islet model at time points (1) and (2) during hub inhibition. Mean (G) ${\left[Ca^{2+}\right]}_{i}$ for all β-cells in a mouse islet model, during recovery from hub inhibition. Raster plot showing ${\left[Ca^{2+}\right]}_{i}$ activity in each β-cell. 3D plot of ${\left[Ca^{2+}\right]}_{i}$ for each β-cell in the islet model at time points (1) and (2). *cf*. S1 Video.

**Figure 2.**
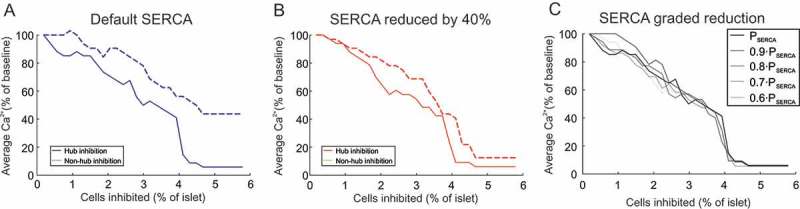
Influence of SERCA on hub importance. (A) ${\left[Ca^{2+}\right]}_{i}$ activity in a mouse islet model as a function of the number of cells inhibited (% of islet). Either hubs or non-hubs were inhibited, and ${\left[Ca^{2+}\right]}_{i}$ activity (% of no inhibition) was quantified. The value of $P_{SERCA}$ (the maximum flux of Ca^2+^ through the SERCA pump) in this model was set to its default value (0.096 amole/ms), according to the Cha-Noma model. (B) Same as in (A) but where $P_{SERCA}$ was reduced by 40%. (C) Same as in (B) but displaying hub inhibition only, with graded reduction in $P_{SERCA}$.

**Figure 3.**
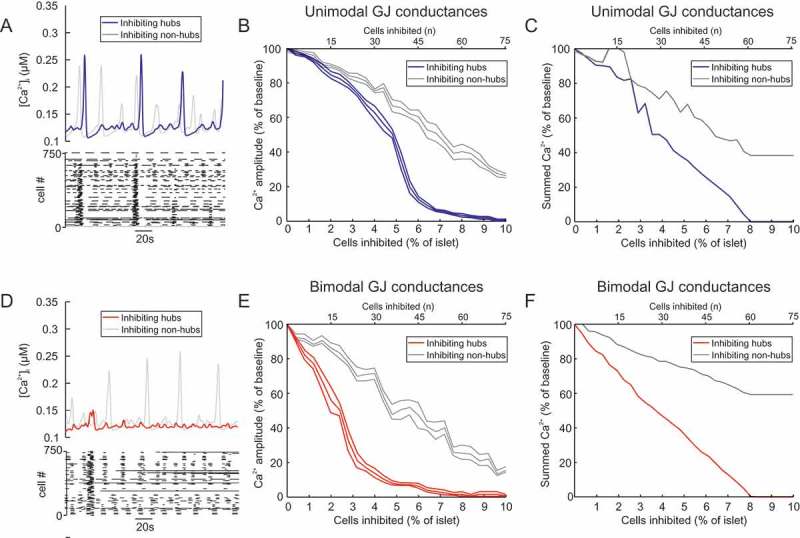
Bimodal gap junction strength influences the importance of hubs. (A) Mean${\left[Ca^{2+}\right]}_{i}$ for all β-cells in a mouse islet model with unimodal GJ conductances (mean 20 pS), during hub inhibition and non-hub inhibition. Raster plot showing ${\left[Ca^{2+}\right]}_{i}$ activity in each β-cell during the hub inhibition condition. (B) ${\left[Ca^{2+}\right]}_{i}$ activity in a mouse islet model as a function of the number of cells inhibited (% of islet). The GJ conductances in this model are unimodal, with GJ conductances for hubs and non-hubs sampled from a distribution with mean 20 pS. Either hubs or non-hubs were inhibited and the resultant ${\left[Ca^{2+}\right]}_{i}$ activity amplitude (% of no inhibition amplitude) was quantified. Error bars show the SEM for re-running of both of these simulations for 6 different random seeds. The hub inhibition simulations have an IC50 of 2.59±0.4% (mean ± SEM). (C) ${\left[Ca^{2+}\right]}_{i}$ activity in a mouse islet model as a function of the number of cells inhibited (% of islet). The GJ conductances in this model are unimodal, with GJ conductances for hubs and non-hubs sampled from a distribution with mean 20 pS. Either hubs or non-hubs were inhibited and the summed ${\left[Ca^{2+}\right]}_{i}$ activity (% of no inhibition) was quantified. (D) Same as in (A) but for all β-cells in a mouse islet model with bimodal GJ conductances, during hub inhibition and non-hub inhibition. Raster plot showing ${\left[Ca^{2+}\right]}_{i}$ activity in each β-cell during the hub inhibition condition. (E) Same as in (B) but for bimodal GJ conductances, with GJ conductances for hubs sampled from a distribution with larger mean (50 pS) than non-hubs (10 pS). Either hubs or non-hubs were inhibited and the resultant ${\left[Ca^{2+}\right]}_{i}$ activity amplitude (% of no inhibition amplitude) was quantified. Error bars show the SEM for re-running of both of these simulations for 6 different random seeds. The hub inhibition simulations have an IC50 of 2.59±0.4% (mean ± SEM). (F) Same as in (B) but for bimodal GJ conductances, with GJ conductances for hubs sampled from a distribution with larger mean (50 pS) than non-hubs (10 pS). Either hubs or non-hubs were inhibited and the summed ${\left[Ca^{2+}\right]}_{i}$ activity (% of no inhibition) was quantified. Note how silencing non-hubs has a minimal effect on summed ${\left[Ca^{2+}\right]}_{i}$ output. *cf*. S2 Video.

**Figure 4.**
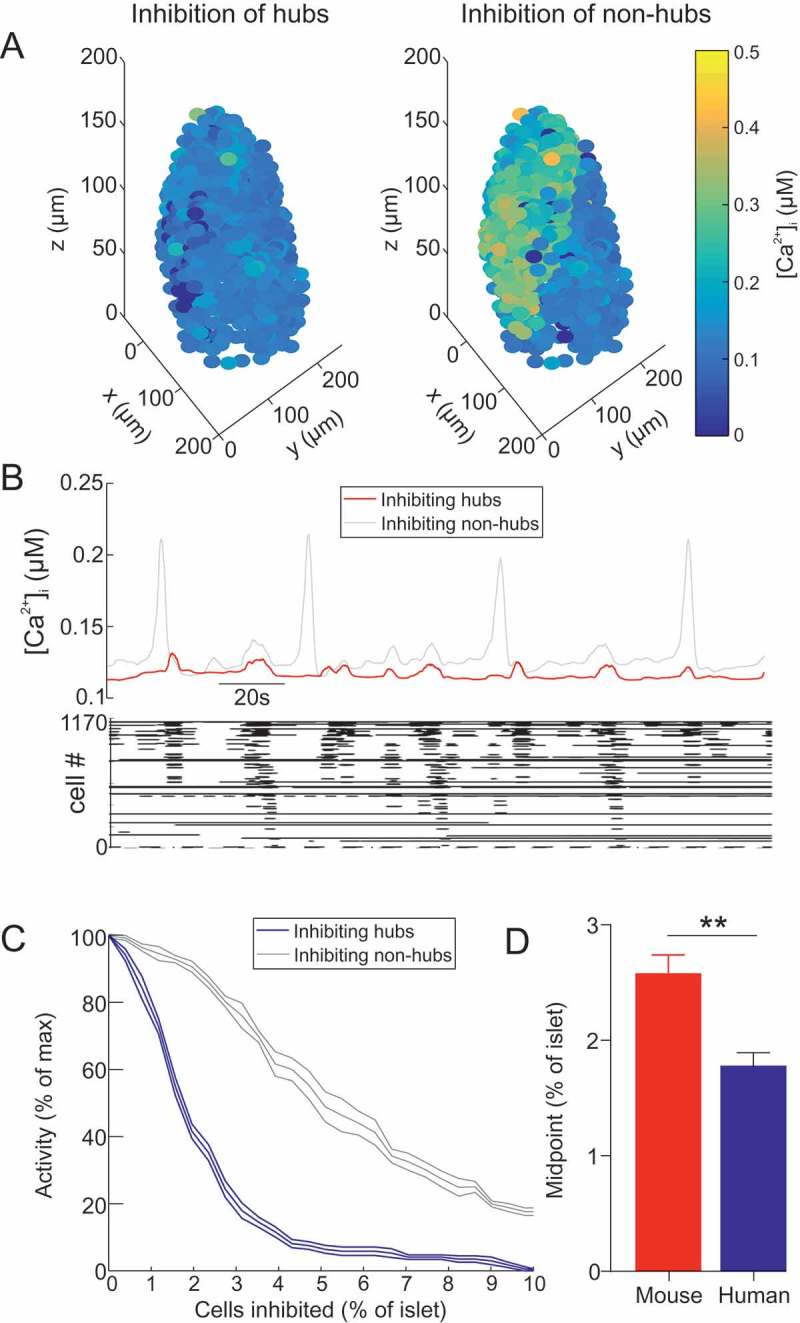
Hub cells dictate whole-islet Ca^2+^ activity in a model of a human islet. (A) 3D plot of ${\left[Ca^{2+}\right]}_{i}$ for each β-cell in a human islet model, during hub inhibition and non-hub inhibition. *cf*. S3 Video. (B) Mean ${\left[Ca^{2+}\right]}_{i}$ for all β-cells in a human islet model, during hub inhibition and non-hub inhibition. Raster plot showing ${\left[Ca^{2+}\right]}_{i}$ activity in each β-cell during the hub inhibition condition. (C) ${\left[Ca^{2+}\right]}_{i}$ activity in a human islet model as a function of the number of cells inhibited (% of islet). Either hubs or non-hubs were inhibited. Error bars show the SEM for re-running of both of these simulations for 6 different random seeds. (D) Comparison of the IC_50_ of (C) in the human islet and mouse islet. Represented as % of hubs (which is 10% of the islet). Unpaired t-test, ** = p < 0.01.

**Figure 5.**
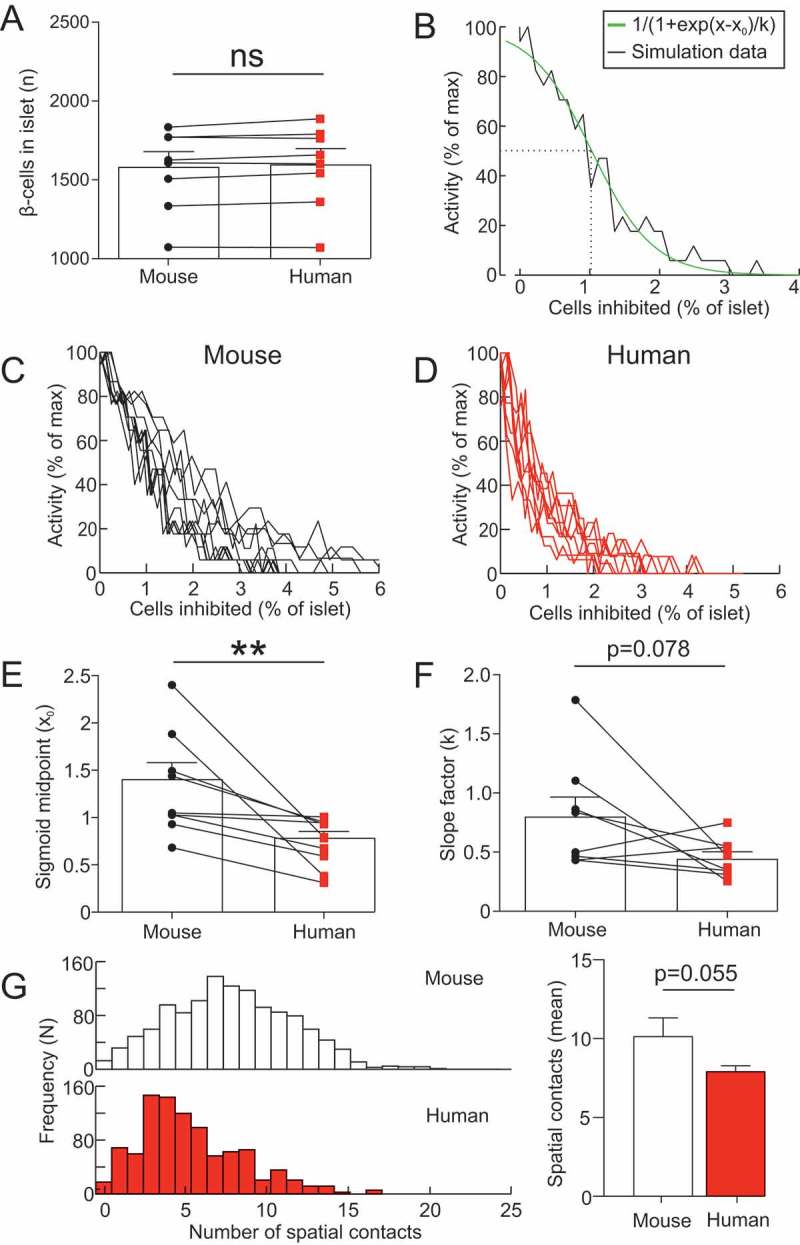
Human islet architectures are more sensitive to hub abolition. (A) Number of β-cells in the 8 mouse islets (4 mice) and 8 human islets (4 donors) used to generate mathematical models of mouse and human islets. Paired t-test, ns = not significant. (B) ${\left[Ca^{2+}\right]}_{i}$ activity in a mouse islet model as a function of the number of cells inhibited (% of islet). This data was fit with a sigmoid function, to determine the IC_50_ $\left(x_{0}\right)$ and slope factor $\left(k\right)$ of the relationship. (C) ${\left[Ca^{2+}\right]}_{i}$ activity in all 8 mouse islet models as a function of the number of cells inhibited (% of islet). (D) ${\left[Ca^{2+}\right]}_{i}$ activity in all 8 human islet models as a function of the number of cells inhibited (% of islet). (E) The IC_50_ $\left(x_{0}\right)$ of the sigmoid function for mouse and human islets. Paired t-test, ** = p < 0.01. (B) The slope factor $\left(k\right)$ of the sigmoid function for mouse and human islets. Paired t-test. (G) The number of spatial contacts between β-cells in mouse (n=8) and human islets (n=8). Two β-cells were deemed in spatial contact if $d_{thr}<17.5$ µm. The distribution of the number of spatial contacts in a mouse and human islet. The mean number of spatial contacts in mouse (n=8) and human (n=8) islets. Paired t-test.

We also considered another situation, whereby the GJ conductance followed a bimodal distribution (see *Defining hubs and non-hubs* below). In both cases, we assumed that the GJ current was linearly related to the difference in membrane potentials, as in previous models of coupled β-cells.^49,50^

### Defining hubs and non-hubs

Johnston et al.^33^ reported that hubs are highly metabolic compared to non-hubs, due to twice the GCK expression. To mimic this doubling of glucokinase expression in hubs, we made hubs more sensitive to glucose. To impose this assumption, we assumed that in simulations of high glucose, glucose for hubs was higher than glucose for non-hubs. In particular, in high glucose, the glucose in hubs was set to 11 mM $\left(\left[G_{hub}\right]=11\right)$, and glucose for non-hubs was set to $\left[G_{non-hub}\right]$. This assumption is a valid representation of this experimental finding, as glucose transport is not rate-limiting for β-cell function.^51^ An appropriate value for $\left[G_{non-hub}\right]$ was to be determined, and was investigated by considering different distributions for $\left[G_{non-hub}\right]$ and comparing the output of the model with the available experimental data. We considered $\left[G_{non-hub}\right]$ to follow a uniform distribution over an interval $\left([G_{non-hub}\right]∼U\left(X,Y\right))$ mM ($U\left(X,Y\right))$, where X and Y were to be determined.

Johnston et al.^33^ reported that hubs constitute 1-10% of the islet. We therefore assumed that 10% of the cells in the islet were highly metabolic (and therefore had a high glucose condition defined by $\left[G_{hub}\right]$). The remaining 90% of cells had a high glucose condition defined by$\;\left[G_{non-hub}\right]$.

Using photo-labeling of hubs, Johnston et al.^33^ demonstrated that hubs have lower protein expression of SERCA2. To explore how this may effect hub and whole-islet function, we explored how altering the flux term for SERCA in the Cha-Noma model ($J_{SERCA}$) influenced the output of the islet model:

(4) $$J_{SERCA}=P_{SERCA}\frac{{{\left[Ca^{2+}\right]}_{i}}^{2}}{{{\left[Ca^{2+}\right]}_{i}}^{2}+K^{2}}$$

Specifically, we reduced the maximal flux ($P_{SERCA}$), which has a default value of 0.096 amole/ms.

Finally, we also considered an addition assumption; that hubs were preferentially GJ coupled to all cells that they are in spatial contact with. The justification for this assumption was as follows:

1. The insulin content of a β-cell is closely related to the extent to which the cell is GJ coupled. In particular, cells with less insulin exhibit larger GJ connectivity.^52,53^ This would suggest that hubs have larger GJ connectivity, as they express less insulin.^33^
2. The extent of ${\left[Ca^{2+}\right]}_{i}$waves in islets is GJ-dependent.^54^
3. Recent data have demonstrated that highly metabolic cells are more efficient at recruiting ${\left[Ca^{2+}\right]}_{i}$ waves in neighboring cells.^55^ As hub cells are highly metabolic,^33^ this would imply that hub cells may be preferentially connected.
4. Recordings of GJ conductances between β-cells in intact islets have demonstrated that the distribution of connectivity is bimodal, with some cells exhibiting stronger connectivity than other cells.^56^

We therefore considered two circumstances:(1) hubs and non-hubs have GJ conductances picked from the same unimodal Gaussian distribution ($N\left(20,14\right)$ pS), and (2) hubs are preferentially GJ coupled. We imposed (2) by introducing a bimodal distribution in the GJ conductance parameter, picking hub GJ conductances from $N\left(10,2\right)$ pS and non-hub GJ conductances from$\;N\left(50,10\right)$ pS.

### Cell-to-cell heterogeneity and parameter uncertainty

β-cells are known to exhibit highly heterogeneous electrophysiological properties.^57-59^ Within an islet model, we picked all maximal conductance parameter values, for both hubs and non-hubs, from a normal distribution with mean value μ equal to original parameter value given in Cha et al.^43^ and standard deviation equal to 20% of the mean value $\left(\sigma =0.2\mu \right)$. This allowed us to account for variability and parameter uncertainty in our simulation results.

### Simulation protocol

To mimic the experimental condition used in Johnston et al.,^33^ islet models were simulated under high glucose, followed by inhibition of a selected population of cells (hubs or non-hubs), and then recovery. Inhibition of β-cells was achieved by clamping the membrane at −100 mV, mimicking the optogenetic silencing of β-cells observed in Johnston et al.^33^

All models were coded in the simulation environment NEURON under the Python interface using CVODE and a 25 µs time-step.^60^ Simulations were conducted in parallel using MPI for Python (mpi4py) on ARCUS-B (Advanced Research Computing, University of Oxford). Simulation of an islet with ~1000 β-cells for 200 sec took ~10 hours. Simulation videos are provided as supplementary material and simulation code is available on a GitHub repository https://github.com/chonlei/bHub_sim

### Analysis of data

All data were imported into MATLAB v6.1 (2000; The MathWorks, Natick, MA, USA) for plotting and analysis. For all β-cells in a simulated islet, the average ${\left[Ca^{2+}\right]}_{i}$ response during each experimental condition was quantified (e.g. during hub silencing). Raster plots were generated of the ${\left[Ca^{2+}\right]}_{i}$data by the same method described by Johnston et al.^33^ In particular, the ${\left[Ca^{2+}\right]}_{i}$signal for each β-cell was binarized by using a threshold (0.2 µM; 40% of maximal ${\left[Ca^{2+}\right]}_{i}$ following data normalization). ${\left[Ca^{2+}\right]}_{i}$activity was represented as a function of the number of hubs (or non-hubs) inhibited, and fit with a sigmoid function (all R^2^>0.9). ${\left[Ca^{2+}\right]}_{i}$ activity was quantified as either the summed ${\left[Ca^{2+}\right]}_{i}$ activity or the maximum amplitude of ${\left[Ca^{2+}\right]}_{i}$ activity, as a function of ${\left[Ca^{2+}\right]}_{i}$ activity under the baseline (no inhibition) condition.

All results are reported as mean ± SEM. Statistical significance was defined as p < 0.05. All statistical tests were conducted in Prism 7.02 (GraphPad Software, San Diego, CA, USA). Significance was assessed with a paired or unpaired *t* test.

## Results

### Exploration of how to define non-hubs in islet models

A model of a mouse islet was constructed and simulated under high glucose conditions (Figure 1, S1 Video). The islet model consisted of 750 β-cells, with 10% hub cells. We first explored in this model how non-hubs should be defined in order to best recapitulate the experimental data of Johnston et al.^33^ We did this by comparing the model output for different distributions over $\left[G_{non-hub}\right]$ to available experiment data from Johnston et al.^33^; in particular, that inhibition a single hub cell in a population of ~50-100 imaged cells (1-2% of the imaged β-cells) could abolish ${\left[Ca^{2+}\right]}_{i}$ activity in the network. To explore how to define $\left[G_{non-hub}\right]$ for non-hubs, we sampled $\left[G_{non-hub}\right]∼U\left(X,Y\right)$, for different intervals $\left[X,Y\right]$ (Figure 1A-D). When $\left[G_{non-hub}\right]∼U\left(6.5,7.5\right)$, the islet produced robust, islet-wide oscillations in ${\left[Ca^{2+}\right]}_{i}$ (Figure 1A). However, inhibition of a large fraction of hubs (IC_50_ = 7.6% of all β-cells in the islet) was required to silence the ${\left[Ca^{2+}\right]}_{i}$ oscillations (Figure 1C and D). This is not in agreement with the experimental data of Johnston et al.^33^ If $\left[G_{non-hub}\right]∼U\left(6,7\right)$, the islet also produced robust ${\left[Ca^{2+}\right]}_{i}$ oscillations (Figure 1B). Moreover, the model better recapitulated the experimental data of Johnston et al.^33^; whole-islet ${\left[Ca^{2+}\right]}_{i}$ activity was strongly suppressed by inhibition of a small number of hubs (IC_50_ = 4% of all β-cells in the islet). We therefore defined $\left[G_{non-hub}\right]∼U\left(6.0,7.0\right)$, because this produced an islet model which best adheres to the experimental data. This distribution over $\left[G_{non-hub}\right]$ was adopted for all islet models from hereon in. This interval includes the Hopf point for initiation of firing (6.9 mM), for the original (non-Gaussian-sampled) parameter values.^61^

### Inhibition of hub cells can abolish whole-islet Ca^2+^ activity

A model of a mouse islet was simulated under high glucose conditions before (Figure 1E), during (Figure 1F) and after (Figure 1G) inhibition of hubs or non-hubs (see also S1 Video). Under basal conditions, the islet model generated highly synchronized ${\left[Ca^{2+}\right]}_{i}$ oscillations (Figure 1E). Simultaneous inhibition of a proportion of these hubs (6% of all β-cells in the islet) severely disrupted these ${\left[Ca^{2+}\right]}_{i}$ oscillations (Figure 1F). Inhibition of the same number of non-hubs was not able to induce such a widespread disturbance to ${\left[Ca^{2+}\right]}_{i}$ activity. Removing this inhibition quickly restored the ${\left[Ca^{2+}\right]}_{i}$ activity (Figure 1G).

### Reducing SERCA does not increase the importance of hubs

In addition to being highly metabolic, Johnston et al.^33^ reported that hubs have reduced SERCA. Therefore, on top of our highly metabolic definition, we next explored the behavior of our model when SERCA was reduced in hubs (Figure 2). Specifically, we reduced the uptake of Ca^2+^ by SERCA into the ER in the model by reducing the maximal flux parameter $P_{SERCA}$. We ran a model wherein hubs were just highly metabolic (Figure 2A) and compared it to a model where hubs were highly metabolic and had 40% reduced SERCA (Figure 2B). The added definition of reduced SERCA did not enhanced the influence of hubs on whole-islet Ca^2+^ activity. In fact, it increased the influence of non-hub silencing on whole-islet Ca^2+^ activity (Figure 2B). As we reduced SERCA in a graded fashion (from 100% to 60% of the default value), there was no obvious improvement in the influence of hub inhibition on whole-islet Ca^2+^ activity (Figure 2C). For this reason, in what follows we did not impose this extra definition of hubs.

### Hubs are preferentially gap junction coupled in the islet network

Although these results demonstrate that a small proportion of hub cells are crucial to whole-islet ${\left[Ca^{2+}\right]}_{i}$ activity, they do not entirely conform to the experimental results of Johnston et al.^33^ In particular, Johnston et al.^33^ reported that inhibiting a single hub cell in a population of ~50-100 imaged cells (1-2% of the imaged β-cells) could abolish ${\left[Ca^{2+}\right]}_{i}$ activity in the network. In contrast, our simulations required inhibition of ~6% of the islet cells to completely silence the islet (Figure 1D). We therefore investigated how to improve the model fit to the experimental data. We postulated that the model may better adhere to the experimental data if hubs had strong GJ connectivity. We therefore explored a situation whereby hub cells exhibited either the same GJ connectivity as non-hub cells (‘unimodal’), or stronger GJ connectivity than non-hub cells (‘bimodal’; Figure 3, S2 Video). In the unimodal case, inhibiting hubs was ineffective at silencing the islet until > 5% of the islet was silenced (Figure 3A-C). Furthermore, silencing non-hub cells had a similar effect on whole-islet ${\left[Ca^{2+}\right]}_{i}$ activity. These results do not conform to the results of Johnston et al.^33^ For bimodal GJ connectivity, the influence of hubs on whole-islet ${\left[Ca^{2+}\right]}_{i}$ activity was greatly increased (Figure 3D-F). The IC_50_ (half-maximal inhibition) was 2.6±0.4% β-cells (Figure 3E), compared to 5.2±0.4% for unimodal GJ heterogeneity (p=0.001, Figure 3B). Furthermore, inhibition of non-hubs was less effective at ceasing whole-islet ${\left[Ca^{2+}\right]}_{i}$ activity (Figure 3D-F). Therefore, assuming that hubs are preferentially GJ connected improves the adherence of the model to the experimental data of Johnston et al.^33^ As a result, we adopt this bimodal GJ assumption from here on in. More importantly, these simulation data suggest that hubs are preferentially GJ coupled in the islet network.

### Hub cells dictate whole-islet Ca^2+^ activity in a model of a human islet

We next sought to investigate whether the results of Johnston et al.^33^ could be recapitulated in a model of a human islet (Figure 4, S3 Video). The human islet model consisted of 1173 β-cells with 10% hubs. When the hubs where inhibited, whole-islet ${\left[Ca^{2+}\right]}_{i}$ activity was severely disrupted (Figure 4A-C). This contrasted with inhibition of non-hubs, which did not disturb whole-islet ${\left[Ca^{2+}\right]}_{i}$activity (Figure 4A-C). These results were not dependent on the random seed for the generation of the parameter values (Figure 4C). Furthermore, the IC_50_ of inhibition of the islet was 1.79±0.3% hub cells - significantly less than in the aforementioned mouse islet model (p=0.003; Figure 4D). Therefore, the human islet model was more sensitive to hub inhibition than the mouse islet model.

### Human islet architectures are more sensitive to hub inhibition

To determine whether this result was consistent across different islet architectures, we repeated these simulations for 8 mouse and 8 size-matched human islet architectures (Figure 5). The size of mouse islets (1565±90 β-cells) was not significantly different to human islets (1585±93 β-cells; p=0.12; Figure 5A), demonstrating that the size-matching of the islets was effective. Each islet was endowed with 10% hubs and simulated in high glucose. For each islet, the number of hubs inhibited was progressively increased and ${\left[Ca^{2+}\right]}_{i}$ activity quantified (Figure 5B-E). A sigmoid was fit to these data, for which an IC_50_ and slope factor could be calculated (Figure 5B; R^2^=0.92±0.03). In mouse islets, the IC_50_ was 1.41±0.2%, compared to 0.78±0.8% in human islets (p=0.008; Figure 5E). The slope factor did not differ across species (p=0.078; Figure 5F). When we examined the distribution of the number of spatial contacts between β-cells in human compared to mouse islets, we observed that the mean was larger in mouse (10.2±1.2 contacts) compared to human (7.9±0.3), although this difference was non-significant (p=0.055; Figure 5G). These data suggest that human islet function is more susceptible to hub disruption than mouse islets, and that this is due to species differences in islet architecture.

## Discussion

In this study we used a computational approach to investigate and compare the role of β-cell hubs in generating coordinated Ca^2+^ oscillations in mouse and human islets. The aim of this study was not to demonstrate that hubs are (/are not) a ubiquitous feature of islets. The aim was to employ an appropriate methodology (namely, computational modeling) to investigate (a) the validity of the results of Johnston et al.^33^ and (b) the potential properties of hubs. To do this in an objective manner, we made as few modeling assumptions as possible, picked parameters from distributions to mimic experimental β-cell heterogeneity, repeated simulations for different random seeds and constructed models for a number of different mouse and human islet architectures.

### Importance of hub cells in whole-islet Ca^2+^ oscillations

By using high-powered and parallelized computing, we were able to demonstrate that endowing an islet with a small proportion of highly metabolic hub cells could result in the generation of synchronous Ca^2+^ activity. Furthermore, this activity could be abolished by inhibiting a few of these cells (~2% of all β-cells in the islet), demonstrating that β-cell hubs can dictate the whole-islet Ca^2+^ response to high glucose. However, non-hub silencing was also able to strongly inhibit the islet, which does not adhere to the data of Johnston et al.^33^ For this reason, we explored additional assumptions that may improve the model fit to the available data.

### Hub cells are highly functionally connected

Connectivity between β-cells is heterogeneous, with certain β-cells exhibiting high connectivity.^62-65^ Recent data have demonstrated that highly metabolic cells are more efficient at recruiting neighboring cells.^55^ Upon glucose stimulation, GJ coupling between β-cells increases.^66^ Furthermore, hub cells express less insulin,^33^ and highly GJ coupled cells are known to express less insulin.^52,53^ Taken together, these data suggest that hub cells may be preferentially connected. For this reason, we added the modeling assumption that hubs are favorably GJ coupled in the islet network. In particular, we endowed our model with a bimodal distribution of GJ connectivity, with hubs exhibiting stronger GJ connectivity than non-hubs. Interestingly, this assumption is supported by recordings of GJ conductances between β-cells in intact islets, which demonstrated that the distribution of connectivity is bimodal, with some cells exhibiting stronger connectivity than others.^56^ When we added this assumption, our simulation data better adhered to the results of Johnston et al.^33^ In particular, inhibition of ~3% of the islet was sufficient to silence the islet. These data therefore suggest that hub cells may not just be highly metabolic, but also highly GJ coupled. Although experimental and computational data strongly supports this, further experimental validation/refutation of this assumption could be conducted.

This could, for example, involve FRAP-based monitoring of functionally identified hubs^64^ or laser capture microdissection of hubs^67^ followed by single cell RNA-seq.^68^

Johnston et al.^33^ reported that hubs express less SERCA than non-hubs. Interestingly, adding this assumption to our models did not improve the adherence of the models to the experimental data. These simulation data do not preclude the findings of Johnston et al.,^33^ but instead indicate that a reduction in SERCA protein expression does not simply imply reduced Ca^2+^ uptake into the ER. This computational finding warrants a more detailed investigation into which SERCA properties are altered in hub cells. Computational models would be able to guide such experimental investigations.

### Human islets are more susceptible to hub disruption than mouse islets

Previous simulation studies have shown that clusters of β-cells are robust against significant perturbations to the islet, including changes to the architecture and β-cell loss.^69^ Our results support this finding, as loss of non-hubs in both mouse and human islets failed to strongly influence Ca^2+^ activity. On the other hand, our simulation data did demonstrate that inhibition of a small number of hub cells can greatly impact islet function. Therefore, it is important to understand how hub cells may become disrupted. This is especially pertinent in human islets, as our simulations revealed that human islets are particularly sensitive to hub cell dysfunction.

Human islets are known to consist of clusters of β-cells, whereas mouse islets have a large, highly connected core,^38-40^ an architectural difference that was reflected in our analysis of the number of spatial contacts between β-cells in islets. These structural differences explain why human islets are more susceptible to hub disruption than mouse. Interestingly, the characteristics of islets from mouse models of diabetes are more similar to human islets.^70^ Therefore, the reduced insulin output from these strains may in part result from hubs experiencing a greater demand in an architecture more sensitive to hub disruption.

Johnston et al.^33^ demonstrated that glucotoxic and glucolipotoxic challenges reduced the proportion of hubs in mouse and human islets. Therefore, hubs may be specifically targeted during pro-inflammatory insults. Our simulation data show that this would have far-reaching effects on Ca^2+^ activity, causing termination of whole-islet function. Such hub-specific failure may contribute to T2DM, as hub cells appear to relate to a previously described β-cell population that are susceptible to cell death.^8,9^

### Study limitations

To recapitulate the higher GCK expression and mitochondrial potential in hubs,^33^ we defined non-hubs to have a lower glucose than hubs in our simulations (Figure 1). We explored an appropriate value for this parameter by determining which value produces an islet model that best adheres to the experimental data. We found that picking non-hubs to have a glucose value from a uniform distribution on 6.0-7.0 mM, best reproduced the experimental data from Johnston et al.^33^ However, this does not necessarily adhere to other available experimental data. In particular, the threshold for firing in the Cha-Noma model is ~6.9 mM, so approximately 90% of our non-hubs are silent in high glucose (when in isolation). This is at odds with data from dispersed β-cells, which exhibit a reliable and robust oscillatory response to high glucose.^71^ However, the mechanical and enzymatic process of β-cell dispersion may alter their firing properties, resulting in increased excitability. Furthermore, removing β-cells from their islet environment may release them from paracrine inhibition, increasing their excitability.

We used a model of electrical activity in a mouse β-cell^43^ to construct models of human islets. Although models of human β-cells exist^50,72,73^ which capture the different electrophysiological properties of these cells compared to rodent β-cells^50,72-75^, there is large variability in the quality, function and donor details of human islets.^76-78^ Furthermore, the data that these models are based on uses recordings from dispersed human β-cells that were cultured in media without the addition of any serum.^50,73,74^ It has subsequently been shown that supplementation of islets with serum is essential for preserving islet function.^79^ Given that human β-cells are known to burst,^75,80-83^ and that the Cha-Noma model generates bursting dynamics, we opted to use the Cha-Noma model as a reliable proxy for a human β-cell in our models of human islets. This also afforded a direct comparison of the influence of islet architecture between mouse and human. To ensure our models represented the known cell-to-cell variability, we picked parameter values from Gaussian distributions. This resulted in simulations that could capture the uncertainty in parameter values from human β-cell recordings.

Finally, we note that we compared inhibiting hubs to inhibiting randomly-selected non-hubs, whereas Johnston et al.^33^ compared inhibiting hubs to inhibiting cells with the lowest number of links. This may explain why, in our simulations, inhibiting non-hubs was still relatively effective at inhibiting the islet. However, we were unable to conduct such a simulation, as we were unable to recapitulate the power law property necessary for identification of such (low linked) cells. This may be because (a) the model failed to capture the biological processes necessary to recapitulate this property and/or (b) more sophisticated measure of similarity between cells is required, as has been conducted for pairs of simulated β-cells.^84^ We note that although power law properties have been reproduced in previous *in silico* studies of islets, this was only during the initial phase of the glucose response.^85^ Furthermore, the impact of hub inhibition was not explored in these models.

### Concluding remarks and future directions

In conclusion, we have demonstrated that endowing an islet model with a small proportion of highly metabolic β-cells can recapitulate the findings of Johnston et al.^33^ This computational finding is credible because we made as few modeling assumptions as possible, considered parameter uncertainty and cell-to-cell variability, repeated simulations for different random seeds and constructed models for a number of different mouse and human islet architectures. Our simulations revealed that hubs may be preferentially GJ coupled, allowing them to exert a powerful influence over whole-islet Ca^2+^ activity. GJ coupling between β-cells is essential for islet function.^6,86^ The strength of this coupling decreases with age^87,88^ and in animal models of diabetes.^89,90^ Therefore, our simulations predict that a reduction in GJ coupling would reduce the ability of hubs to generate whole-islet Ca^2+^ oscillations, greatly impairing insulin output. Whether such dysfunctions in hub cells occur in T2DM and contribute to the impaired insulin secretion observed in this disease, remains to be seen. However, simulations of islets will aid our understanding of how these specialized cells contribute to islet function and the aetiology of diabetes.
