# Supplementary material for: Beta-cell hubs maintain Ca2+ oscillations in human and mouse islet simulations
Source: Islets. 2018 Aug 24;10(4):151–67. doi: 10.1080/19382014.2018.1493316 (PMC6113907; doi:10.1080/19382014.2018.1493316)
Supplement: Supplemental Material [file kisl-10-04-1493316-s001.zip › captions to supplementary material.docx]

### S1 Figure: Process for constructing islet models

1. Islets were isolated from mouse (n=4) and human (n=4) donors.
2. Islets were then stained for insulin, and imaged on a 3D confocal microscope. By taking sequential (3-5 µm) z-stack images, the $(x,y,z)$ location of each insulin^+^ cell could be recorded, as described in Hoang *et al.* (36).
3. 3D representation of all β-cells in an imaged islet. This process was conducted for 8 mouse and 8 human islets. The β-cell model of Cha *et al.* (43), as described in Equation (2), was then put at each such $(x,y,z)$ location.
4. To determine whether two β-cells are functionally connected, the Euclidean distance ($d$) between them was calculated.
5. If $d < d_{thr}$ then the cells were functionally coupled via gap junction (GJ). The conductance of this GJ connection was picked from either a unimodal or bimodal Gaussian distribution.
6. If $d \geq d_{thr}$ then the cells were not GJ coupled.

### S1 Video: Inhibition of highly metabolic cells can abolish whole-islet activity

Video of simulation of mouse islet. Islet contains 750 β-cells. Simultaneous inhibition of 10 cells (hubs or non-hubs) occurs at t = 200 sec. *cf.* Figure 1.

### S2 Video: Influence of GJ parameter on hub function

Video of simulation of mouse islet. Islet contains 750 β-cells. Simultaneous inhibition of 22 hub cells (3% of islet) occurs at t = 200 sec. Hub cells either have stronger GJ connectivity, as prescribed by a bimodal GJ distribution, or all cells have the same GJ connectivity (picked from a unimodal distribution. *cf.* Figure 3B, C.

### S3 Video: Hub cells dictate Ca^2+^ activity in human islets

Video of simulation of human islet. Islet contains 1173 β-cells. Simultaneous inhibition of 23 cells (hubs or non-hubs; 2% of islet) occurs at t = 200 sec. *cf.* Figure 4.
