# Supplementary figures and images for: Beta-cell hubs maintain Ca2+ oscillations in human and mouse islet simulations
Source: Islets. 2018 Aug 24;10(4):151–67. doi: 10.1080/19382014.2018.1493316 (PMC6113907; doi:10.1080/19382014.2018.1493316)

# S1 Figure

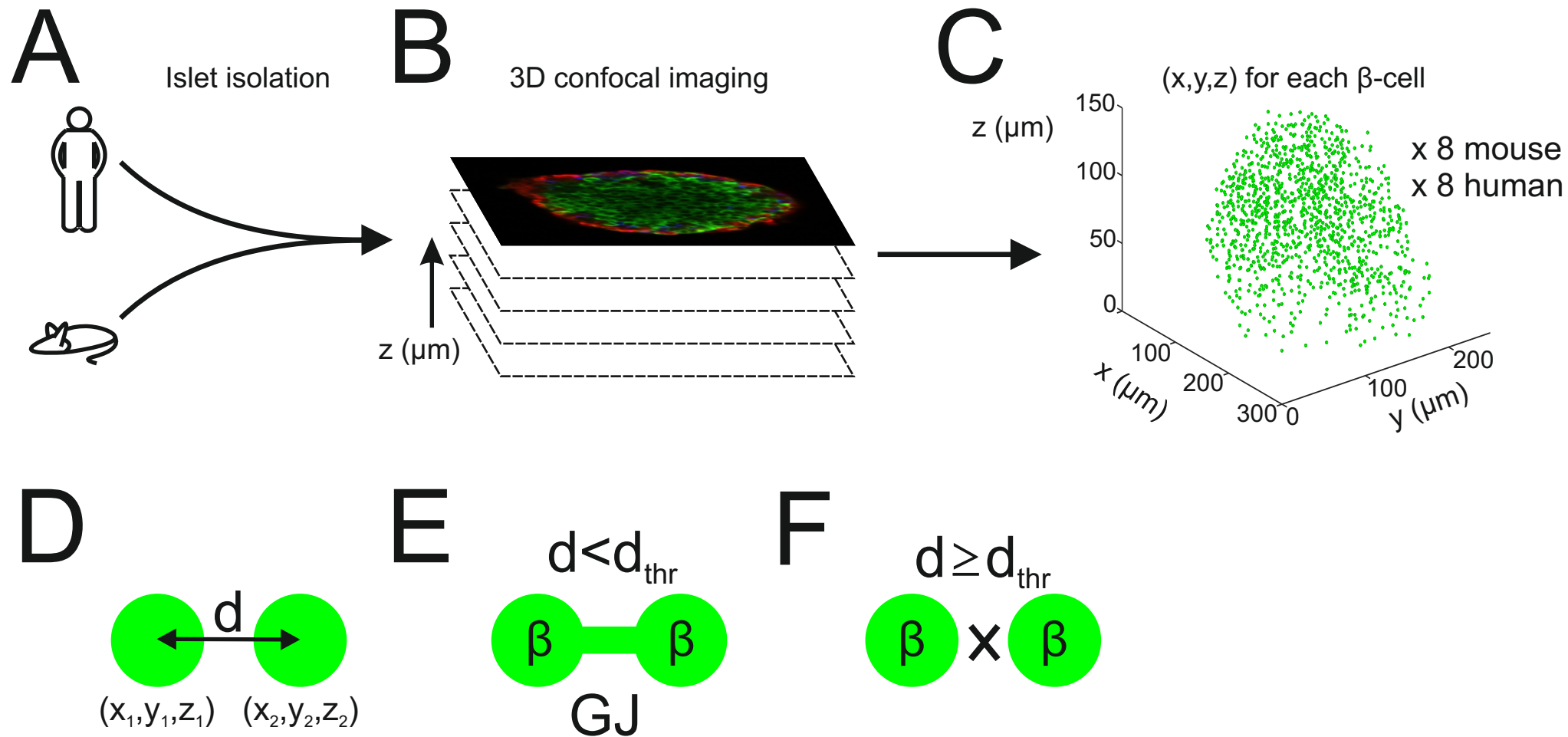

Supplement: Supplemental Material [file kisl-10-04-1493316-s001.zip › S1Figure.pdf]
